# Pistachio Shell-Derived Carbon Activated with Phosphoric Acid: A More Efficient Procedure to Improve the Performance of Li–S Batteries

**DOI:** 10.3390/nano10050840

**Published:** 2020-04-27

**Authors:** Almudena Benítez, Julián Morales, Álvaro Caballero

**Affiliations:** Departamento de Química Inorgánica e Ingeniería Química, Instituto Universitario de Investigación en Química Fina y Nanoquímica, Facultad de Ciencias, Universidad de Córdoba, 14071 Córdoba, Spain; q62betoa@uco.es (A.B.); iq1mopaj@uco.es (J.M.)

**Keywords:** activated carbon, biomass, pistachio shell, porous carbon, lithium–sulfur batteries

## Abstract

A sustainable and low-cost lithium–sulfur (Li–S) battery was produced by reusing abundant waste from biomass as a raw material. Pistachio shell was the by-product from the agri-food industry chosen to obtain activated carbon with excellent textural properties, which acts as a conductive matrix for sulfur. Pistachio shell-derived carbon activated with phosphoric acid exhibits a high surface area (1345 m^2^·g^−1^) and pore volume (0.67 cm^3^·g^−1^), together with an interconnected system of micropores and mesopores that is capable of accommodating significant amounts of S and enhancing the charge carrier mobility of the electrochemical reaction. Moreover, preparation of the S composite was carried out by simple wet grinding of the components, eliminating the usual stage of S melting. The cell performance was very satisfactory, both in long-term cycling measurements and in rate capability tests. After the initial cycles required for cell stabilization, it maintained good capacity retention for the 300 cycles measured (the capacity loss was barely 0.85 mAh·g^−1^ per cycle). In the rate capability test, the capacity released was around 650 mAh·g^−1^ at 1C, a higher value than that supplied by other activated carbons from nut wastes.

## 1. Introduction

Current energy challenges involve overcoming growing energy needs, climate change, and the increasing scarcity of resources. In the search for a competitive, clean, safe, and efficient energy storage system, the development of scientific studies of lithium–sulfur (Li–S) batteries for future commercialization is a key issue for many researchers [1]. Sulfur, the active material of the battery, has the following advantages: (i) It is very abundant in the earth’s crust, is an enormous residue from the petrochemical industry, and is a cheap material; (ii) it is non-toxic and environmentally friendly due to the fact that its production process is well-controlled industrially; and (iii) it is highly energetic in its reversible reaction with lithium [2], being able to supply a theoretical specific capacity of 1675 mAh·g^−1^ (corresponding to an energy density of ~2500 Wh·kg^−1^). However, Li–S batteries must overcome different obstacles to avoid capacity fading during cycling, such as the following [3]: (a) Sulfur and solid reduction products (Li_2_S_2_ and Li_2_S) are insulators, leading to poor electrochemical utilization of the element; (b) formation of intermediate polysulfide, causing severe changes in the volume on the cathode; and (c) dissolution in the electrolyte of some formed polysulfides (Li_2_S*_x_*, 2 ≤ *x* ≤ 8), giving rise to the shuttle phenomenon that causes loss of active material and coulombic efficiency. Several strategies [4,5,6,7] have been investigated to reduce the shuttle effect, such as the use of modified separators [8,9,10,11,12], interlayers [13,14,15], solid-polymer electrolytes [16], or the addition of LiNO_3_ in the electrolyte that passivates the lithium surface and mitigates the problems that take place in the anode. However, the use of porous carbonaceous materials in the positive electrode constitute an excellent matrix for the immobilization of sulfur and entrapment of polysulfides, such as carbon nanotubes [17], carbon nanofibers [18,19,20], carbon flowers [21], graphene [22,23,24,25,26], graphene doped with heteroatoms [27,28], graphene oxide [29], ordered mesoporous carbons [30,31,32], and metal oxide–carbon composites [33]. Unfortunately, most synthetic procedures for these materials are complex and require expensive and non-renewable raw materials, a drawback for large-scale applications.

Porous or activated carbons (ACs) might be an alternative to these more complex systems [34]. In this context, biomass-derived carbon has been widely studied as a promising sulfur host due to its natural abundance, simple and eco-friendly preparation, and low cost. Furthermore, depending on their origin, these easily accessible active carbons have different morphologies and structural properties [35,36]. Thus, a wide variety of waste materials can be used as a matrix to accommodate sulfur, providing a second life to agri-food waste and, at the same time, improving the performance of Li–S batteries, among which it is worth highlighting peanut shell [37], bamboo [38], coconut shell [39], walnut shell [40] with surface hydroxyl groups, or coffee waste with O- and N-doped groups [41]. In addition, their high surface area, large pore volume, and good conductivity have given these materials the proper characteristics to be used as bio-templates to host sulfur and as a separator for preventing the shuttle effect [35].

Our main goal in meeting the challenge of using a sustainable, economical, and renewable source was to research only abundant, nearby, and non-edible sources to improve energy storage systems by reusing waste without having to rely on primary sources. In previous studies, our group revalued the agri-food residues with the highest production in Spain, such as olive stones [42,43,44], cherry pits [45], and almond shells [46]. However, the cultivation of pistachio is becoming very fashionable, and large amounts of shells are currently also generated after consumption. According to the statistics presented by the Food and Agriculture Organization (FAO), in 2018, a total of approximately 1300 kilotons of pistachio were produced in the world, of which 550 kilotons were harvested in Iran (the largest producer in addition to the USA) [47]. During the processing of this crop, a high quantity of residues in the form of shell is generated (equivalent to 45% by weight on average of its production) [48], giving rise to a total of 585 kilotons of pistachio shells in the world during that year. For these reasons, the valorization of pistachio shells (PSs) is crucial to achieving sustainable processing. Thus, PSs were chemically activated with phosphoric acid to obtain AC with optimal textural properties for application in Li–S batteries. Additionally, its porous structure favors penetration of the electrolyte, the entrapment of polysulfides, and the diffusion of ions through the matrix. Herein, we report the electrochemical results obtained using pistachio shell-derived activated carbon (PSAC) as a conductive and renewable matrix. To our knowledge, the use of PSAC in Li–S cells has recently been reported by Chen et al. [49], using activation by ZnCl_2_. This activating agent, together with KOH and H_3_PO_4_, is the most used in the chemical activation of biomass-derived carbons. However, the use of ZnCl_2_ usually involves the formation of ZnO as an impurity, and its elimination requires subsequent treatment with HCl [50]. Here, as an activating agent, we used H_3_PO_4_, which has been preferably used in the activation of many lignocellulosic materials [51]. The reason for this is the ability of H_3_PO_4_ to generate P-containing functional groups in the carbon matrix, which are suitable for inducing the formation of micropores in the carbon, beneficial for use in electrochemical energy storage devices [52]. We previously reported the use of H_3_PO_4_ to activate carbon derived from cherry pits and its application in a Li–S battery [45]. The performance of the electrode when the pistachio carbon was activated with H_3_PO_4_ was notably better than that already reported for PSAC activated with ZnCl_2_. The presence of impurities and possibly the different pore systems generated by both agents might be the cause of the differences found.

## 2. Materials and Methods 

### 2.1. Material and Electrode Preparation

Fresh pistachio shells generated in Spanish crops were used in this study, without any prior treatment. Firstly, pistachio shells were ground in a ball mill (Restch PM100, Retsch GmbH, Haan, Germany) at 500 rpm for 4 h, with reversal of rotation every 15 min. Once a fine powder was obtained, the residue was treated with phosphoric acid (85%, Sigma-Aldrich, San Luis, CA, USA). This treatment was carried out by mixing PS and H_3_PO_4_ in a 1:1 ratio by weight. In detail, 10 g of PS were added to 40 mL of a 3 M H_3_PO_4_ aqueous solution, and this mixture was maintained under constant stirring at 85 °C until a paste was formed in approximately 3 h. Subsequently, the sample was dried at 120 °C overnight, ground in a manual mortar, and pyrolyzed in a tubular furnace (Carbolite Gero CTF, Parsons Lane, Hope Valley, UK) at 800 °C under a nitrogen atmosphere using a flow of 50 mL·min^−1^ for 4 h to ensure successful carbonization of this material, as was demonstrated in our previous work [46]. Later, the pyrolyzed sample was washed several times with distilled water until obtaining a neutral pH and dried again at 120 °C for 12 h. After homogenization of the sample, it was denoted as PSAC. 

The second step was the preparation of the activated carbon/sulfur composite (PSAC@S) using a simple and easily scalable process. A planetary ball mill (Restch PM100, Retsch GmbH, Haan, Germany) was used to mix PSAC and sulfur in a mass ratio of 3:7 with ethanol as a solvent. The wet milling process was carried out at 300 rpm for 3 h, without rest time, to achieve excellent sulfur impregnation. After this process, the obtained product was dried at 50 °C overnight and then ground in a mortar.

**Scheme 1:**
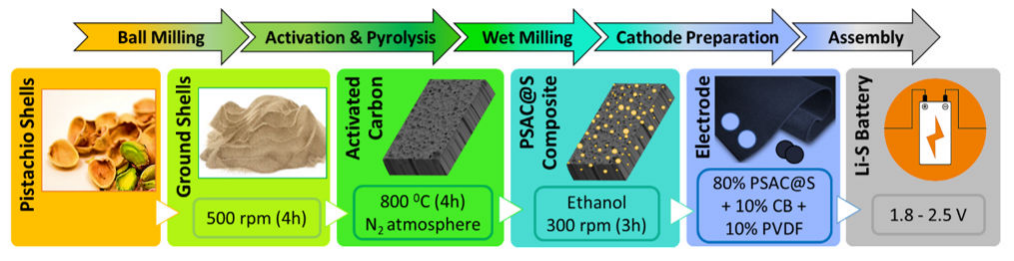
Schematic diagram for the preparation of pistachio shell-derived activated carbon/sulfur (PSAC@S) composite as cathode for lithium–sulfur (Li–S) batteries.

Finally, the positive electrodes were prepared by mixing 80 wt.% of the as-prepared PSAC@S composite, 10 wt.% of the conductive agent (Super P carbon, Timcal, Bironico, Switzerland), and 10 wt.% of polyvinylidene fluoride (PVDF 6020, Solvay, Brussels, Beligum) as a binder in N-methyl-2-pyrrolidone (NMP, Sigma-Aldrich) in a manual mortar until a homogeneous slurry was formed. Then, the slurry was casted on a carbon cloth (GDL, ELAT LT1400W, MTI Corp., Richmond, USA) as a current collector by the doctor blade (MTI Corp.) technique. GDL carbon cloth has been reported as an effective current collector for carbon–sulfur composites of Li–S cells [53]. After drying, the electrodes were punched into 13 mm diameter discs (1.33 cm^2^ geometric surface). Subsequently, the cathode discs were further dried in a vacuum oven (Buchi, Flawil, Switzerland) at 45 °C for 3 h to guarantee the absence of oxygen and humidity in the electrodes. The sulfur loading was ca. 2.5 mg·cm^–2^. The diagram shown in Scheme 1 summarises the sequential steps used for the preparation of the Li–S cells based on pistachio shell-derived carbon/sulfur composite.

### 2.2. Material Characterization

The structural and textural properties of the as-fabricated materials were analyzed by different characterization techniques. X-ray diffraction (XRD) was used to study the structures, and the patterns were obtained through a Bruker D8 Advance diffractometer equipped with a Cu Kα source (λ = 1.5406 Å) at 40 kV and 40 mA over the 2*θ* range of 5° to 80° at a rate of 1.05 s per step with a step size of 0.04°. Raman spectra were recorded with a confocal Raman spectrometer (alpha500, WITec, Ulm, Germany). A frequency-doubled Nd:YAG laser at 532 nm (second harmonic generation) was used for excitation. The laser beam was focused on the sample using a 20×/0.4 Zeiss objective. Thermogravimetric analysis was carried out using a Mettler Toledo-TGA/DSC with a heating rate of 10 °C min^−1^ under N_2_ atmosphere in the range between 30 and 1000 °C for the sample of fresh pistachio shell, in a N_2_ or O_2_ atmosphere from 30 to 800 °C for PSAC and in a nitrogen atmosphere in the range of 30–600 °C for the PSAC@S composite. A nitrogen adsorption–desorption isotherm was obtained on an Autosorb iQ/ASiQwin (Quantachrome Instruments, Anton Paar GmbH, Graz, Austria). Specific surface areas were determined by the Brunauer–Emmett–Teller (BET) method. Total pore volumes (*V_T_*) were determined according to the amount adsorbed at a relative pressure (*P/P_0_*_)_ of 0.995. Pore size distributions were calculated by the density functional theory (DFT) method applied to the adsorption branch of the isotherms. The micropore surface area and volume were estimated by the t-plot method. 

### 2.3. Cell Assembly and Electrochemical Measurements

To explore the electrochemical behavior of the working electrode, coin-type CR2032 cells were assembled inside an Ar-filled glove box (M-Braun 150, M-Braun, Garching, Germany). Lithium metal foil (Li, Gelon Lib, Qingdao, China; 15.6 mm diameter and 0.25 mm thickness) served as the counter and reference electrodes. A polyethylene membrane (PE, 25 µm thick, Celgard 2400, Charlotte, NC, USA) was used as a separator, and it was soaked in 50 μL of the electrolyte solution and dried at 80 °C under vacuum overnight prior to use. The electrolyte was 1 M LiTFSI with 0.4 M LiNO_3_ in in 1,3-dioxolane (DOL, Sigma Aldrich) and 1,2-dimethoxyethane (DME, Sigma Aldrich) (1:1 v/v). The electrochemical process was studied by cyclic voltammetry (CV) and electrochemical impedance spectroscopy (EIS) measurements, which were performed using the Autolab PGSTAT-204 (Metrohm, Herisau, Switzerland) equipment. In order to calculate the lithium-ion diffusion coefficients, additional CV measurements were recorded at variable rates of 0.1, 0.2, 0.4, 0.8, and 1.0 mV·s^−1^ between 1.8 and 2.8 V vs. Li/Li^+^. EIS measurements were taken at the open-circuit voltage (OCV) and after five CV cycles in the 500 kHz to 100 mHz frequency range using a 10 mV amplitude signal. Galvanostatic cycling tests were performed on an Arbin BT2143 (Arbin Instruments, College Station, USA) within the potential window of 1.8–2.6 V at a constant current of C/10 (1 C = 1675 mA·g_S_^−1^) over 300 cycles. Rate capability tests were performed at C/10, C/8, C/5, C/3, C/2, 1C, and 2C rates. 

### 3. Results and Discussion

**Figure 1:**
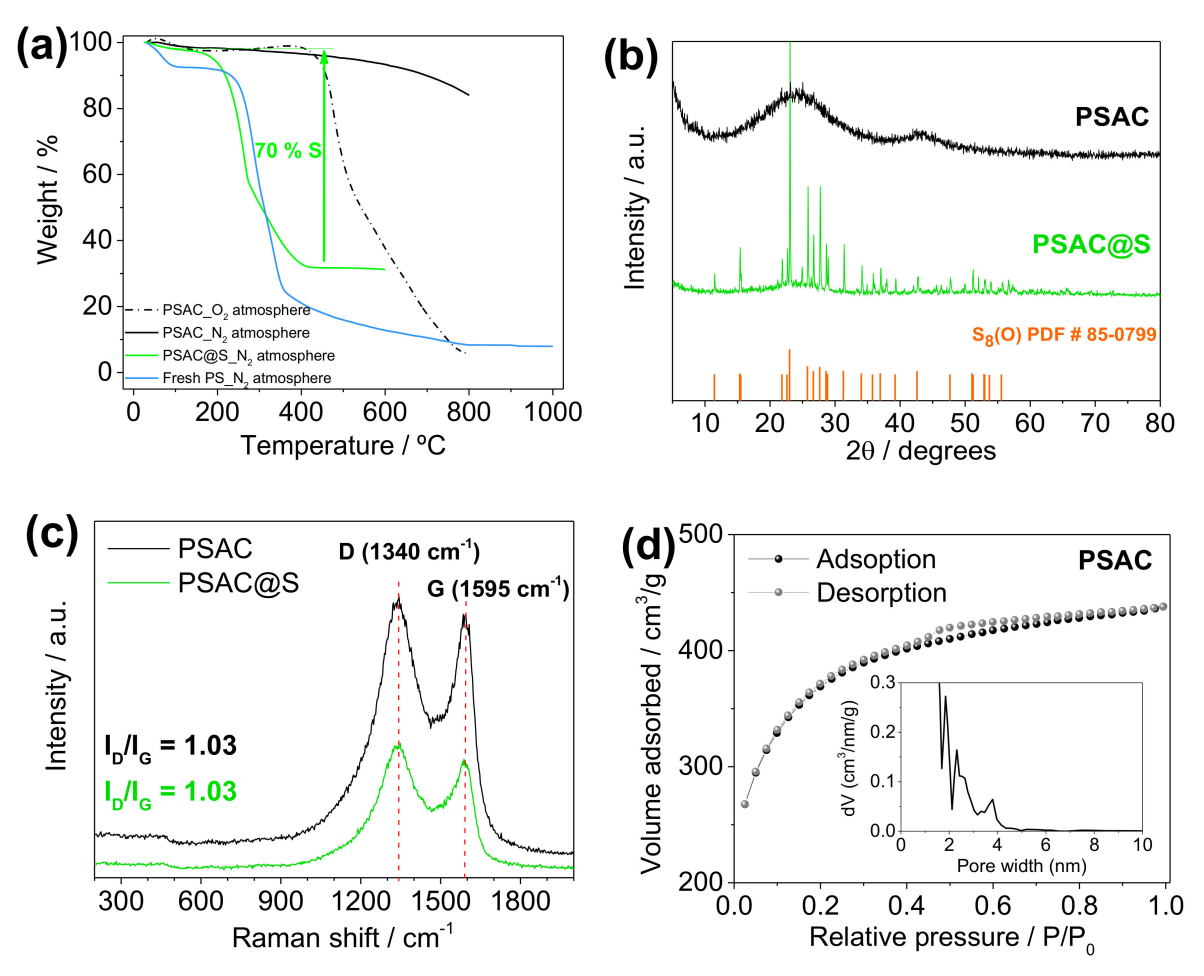
(**a**) Thermogravimetric analysis (TGA) curves of fresh pistachio shell (blue line) in a nitrogen atmosphere, pistachio shell-derived activated carbon (PSAC) in oxygen (dotted black line) and nitrogen (black line) atmospheres, and PSAC@S composite (green line) in a nitrogen atmosphere. (**b**) X-ray diffraction (XRD) patterns and (**c**) Raman spectra of PSAC and the PSAC@S composite. (**d**) N_2_ adsorption/desorption isotherm and pore size distribution of PSAC.

Knowledge of the carbon composition was obtained from thermogravimetric measurements carried out under different atmospheres (Figure 1). To prepare the activated carbon, a temperature of 800 °C was selected according to the thermogram registered for the fresh pistachio shell in inert atmosphere (Figure 1a). The weight loss values of AC (Figure 1a) were 94% and 14% when heated to 800 °C in an O_2_ and N_2_ atmosphere, respectively. This means that both the ash and functional group content was low and, consequently, the impurity content was relatively low. The functional groups presented in these H_3_PO_4_-activated carbons obtained from biomass [54], mainly associated with O and H, would be responsible for the slowness in the weight loss observed (between 400 and 800 °C) compared to the highly graphitized carbons [55] or the graphite itself [56], the weight loss of which is more abrupt and occurs between 550 and 700 °C. The high degree of carbon structural disorder was demonstrated by two complementary techniques: XRD and Raman spectroscopy. The XRD pattern exhibited two broad, weak peaks, typical of highly disordered carbons (Figure 1b). The spacing of the (002) reflection is around 3.68 Å, higher than 3.44 Å, a limit value that allows calculation of the graphitization degree of C [57]. As these carbons are hard carbons, this model would not be applicable. As expected, the XRD of the PSAC@S composite exhibited numerous peaks, all assignable to the S orthorhombic phase. The C signal was barely noticeable because it was found as a minor component. Figure 1c shows the Raman spectra of the PSAC and PSAC@S composite recorded between 200 and 2000 cm^−1^. Two characteristic peaks could be found for both samples. The D band at 1340 cm^−1^ can be assigned to the defects and disorder [58], while the G band at 1595 cm^−1^ corresponds to the presence of sp^2^ hybridized carbon [59]. This is a typical characteristic of the activated carbon [60]. The peak height ratio of *I_D_/I_G_* was 1.03, indicating that the graphitization phase plays a big role in the as-prepared carbon from the pistachio shell [61], which can promote the electrical conductivity of PSAC [62]. After sulfur permeation, the *I_D_/I_G_* ratio value remained unchanged, indicating that the structure of the activated carbon was modified during the melt-diffusion process. The Raman spectrum of the PSAC@S composite showed no characteristic peaks of sulfur at approximately 200 and 460 cm^−1^, which implies that crystalline sulfur was successfully embedded into the pore structure of carbon material. The absence of sulfur peaks may be due to interferences of carbon micro-mesopores in the Raman signals of encapsulated S [63], as similarly reported for other sulfur-based composites with highly porous biomass-derived carbons [39,64].

The S content in the PSAC@S composite was obtained from TGA measurements (Figure 1a). Up to around 160 °C, weight loss is attributed to the physisorbed and chemisorbed solvents (e.g., residual water and ethanol). The remainder, around 70%, would correspond to S sublimation. This loss occurs in two steps, as suggested by the change in slope at ca. 273 °C. This behavior is similar to that found in other microporous materials, such as metal–organic frameworks (MOFs) [65] and in ZnCl_2_-activated pistachio carbon itself [49]. This means that there would be two types of interactions between S and the host matrix. Some authors [65] have suggested that S sublimated at temperatures below 273 °C would be physisorbed S and chemisorbed S of a higher temperature. In the case of microporous carbons, there is an alternative explanation, assigning the loss at low temperature to S deposited on the external surface of C. From 273 °C, nanoparticles confined in the micropores would sublimate [66]. In both cases, additional measurements are necessary to verify these models. 

The N_2_ adsorption/desorption isotherms are shown in Figure 1d. The shape of the adsorption isotherm is typical of microporous solids belonging to type I of the BDDT classification. The presence of a small hysteresis loop in the desorption curve would be due to the presence of mesopores in the C texture. Hence, the PSAC sample possesses a dual pore system that is beneficial both for electrolyte impregnation and for the mobility of Li^+^ ions between the electrodes. The BET surface was 1345 m^2^ g^–1^, and the total pore volume was 0.67 cm^3^·g^−1^. The *t-*plot analysis indicated that a significant fraction of the carbon surface area (973 m^2^·g^−1^) and pore volume (0.4 cm^3^·g^−1^) was associated with the presence of micropores. Regarding the size of the pores, values of 1.6–3.8 nm were deduced from the DFT representation, indicative of the expected dual pore system. The surface of our carbon was greater than that obtained by Chen et al. [49] (1149 m^2^·g^−1^). Although the value of the total pore volume was not given, the pore size was less than 2 nm, even though the representation seems to correspond to the application of the Joyner–Barret–Halenda (JBH) method, which is applicable to mesoporous solids. The differences could be due to both the different activator used, ZnCl_2_, and the heating temperature, 550 °C.

**Figure 2:**
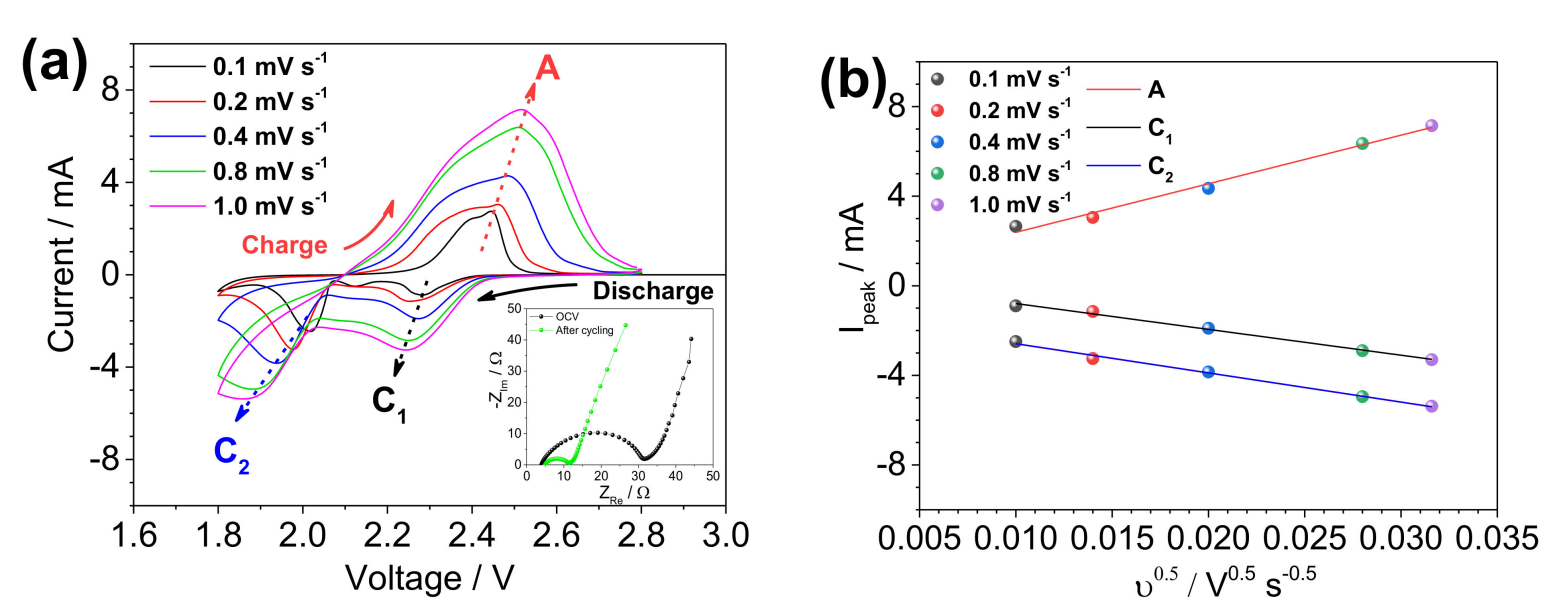
(**a**) Cyclic voltammograms recorded at different scan rates (from 0.1 to 1.0 mV·s^–1^). Electrochemical impedance spectroscopy (EIS) spectra before (black) and after (green) the first cycle, recorded at 0.1 mV·s^−1^, are shown in the inset. (**b**) Variation in the peak current with the square root of the scan rate (Randles–Sevcik equation).

Cyclic voltammograms recorded at different scan rates (0.1–1 mV·s^−1^) are shown in Figure 2a. The common feature is the presence of two peaks during the cathodic scan (2.34 and 1.99 V at 0.1 mV·s^–1^), caused by the reduction of S to high- (Li_2_S*_x_*, 4 ≤ *x* ≤ 8) and low-order (Li_2_S_2_ and Li_2_S) polysulfides, respectively. The reaction mechanism has been thoroughly investigated by applying the operando X-ray diffraction and Raman spectroscopy [67,68,69]. The corresponding oxidation peaks appeared at about 2.39 and 2.44 V due to the slight hysteresis of the reversible redox reaction [70,71,72]. As expected, there were small shifts in the peak potential due to the change in scan rate, which form the basis of the Randles–Sevcik equation for calculating the diffusion coefficient of the Li^+^ ion, as follows:

*I_P_* = 2.69 ×10^5^*n^3/2^ A D*_Li_^0.5^*υ*^0.5^*C*_Li_ (1)

where *I*_P_ is the peak current, *n* is the number of electron transfers in the reaction, *A* is the electrode geometric area, *υ* is the scan rate, and *C*_Li_ is the concentration of lithium ions in the electrolyte. The relationship of *I_P_* vs. *υ*^0.5^ is shown in Figure 2b. From the slope of these plots and Equation (1), the *D*_Li_ values of the two cathodic peaks were 1.6 × 10^–9^ (*C*_1_) and 2.6 × 10^–10^ (*C*_2_) cm^2^·s^–1^, and of the anodic peak, the value was 5.8 × 10^–9^ (*A*) cm^2^·s^–1^. These values are comparable to those calculated for other systems [73,74] and somewhat lower than those obtained in a semi-liquid cell using a Li_2_S_8_ catholyte instead of S as the active material [75].

To shed some additional light on the electrochemical process, EIS spectra of the cell were recorded before and after the 1st cycle. The Nyquist plots are shown in Figure 2a, and, as expected, a similar value for the electrolyte resistance (*Re*), between 4 and 5 Ω, was deduced from the cut of the x-axis at high frequencies. At high-medium frequencies, a semicircle was observed, related to the electrode/electrolyte interphase, the resistance of which was around 27.2 and 6.5 Ω before and after cycling, respectively. This decrease has been observed in other works [71,74] and has been ascribed to the proper change in the electrode due to cycling. Once the electrode is activated, the low resistance value is consistent with both suitable mobility of the charge carriers at the electrode/electrolyte interphase and satisfactory kinetics of the commented redox reactions. The behavior of our activated carbon was somewhat different from that reported by Chen et al. [49], where carbon was activated with ZnCl_2_. The Nyquist plot profile for the pristine electrode was similar but with an electrode resistance somewhat higher (40 Ω). However, a more complex Nyquist plot was obtained for the cycled cell. In the high-medium frequency region, two semicircles with resistances of around 31 and 41 Ω were observed. These data are simply an example of the many factors that affect the electrochemical reaction. When the pistachio carbon is not activated [56], the EIS spectrum is more complex, considerably increasing the resistance of the interface, approaching 100 Ω. In addition, this resistance increases as the electrode is cycled.

**Figure 3:**
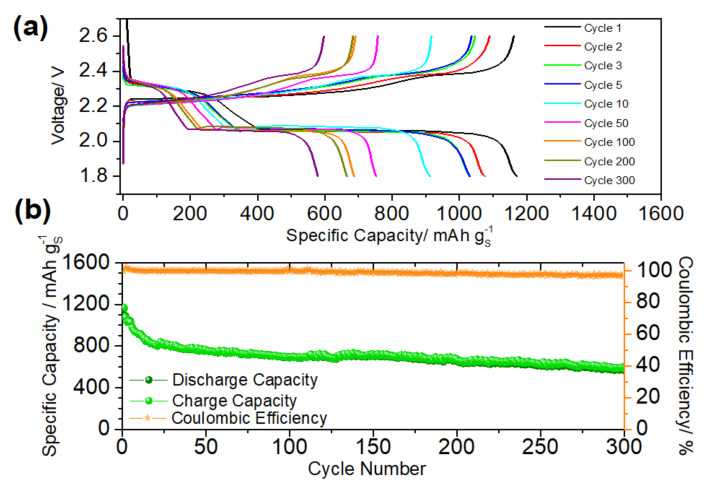
(**a**) Galvanostatic charge/discharge curves at different cycles recorded at *C*/10. (**b**) Variation in the discharge capacity and coulombic efficiency as a function of the number of cycles.

Figure 3a shows the charge/discharge curves for different cycles recorded at *C*/10 (*C* = 1675 mA·g^−1^) between 2.6 and 1.8 V. Below this voltage, another reduction process begins, probably associated with the electrolyte decomposition. Hence, we have chosen to limit the voltage window at the detriment of the specific capacity values. The two plateaus of the discharge curves, ca. 2.31 and 2.06, reflect the two stages of the conversion of S into Li_2_S_2_/Li_2_S, as was previously discussed for the *CV* curves. The charge curves also show two plateaus with less polarization between them, corresponding to the inverse reaction (origin of the peak broadening observed for the *CV* curve). The cycling properties of the electrode are shown in Figure 3b. The delivered capacity in the first discharge, 1193 mAh·g^−1^, sharply decreased in the first twenty cycles to 808 mAh·g^−1^. From this cycle, the capacity retention significantly improved, and at the 300th cycle, the last cycle tested, the retention capacity was 570 mAh·g^−1^, which means an average capacity loss of 0.85 mAh·g^−1^ per cycle. The fluctuation in the coulombic efficiency values was less, with an average value of around 98%.

**Figure 4:**
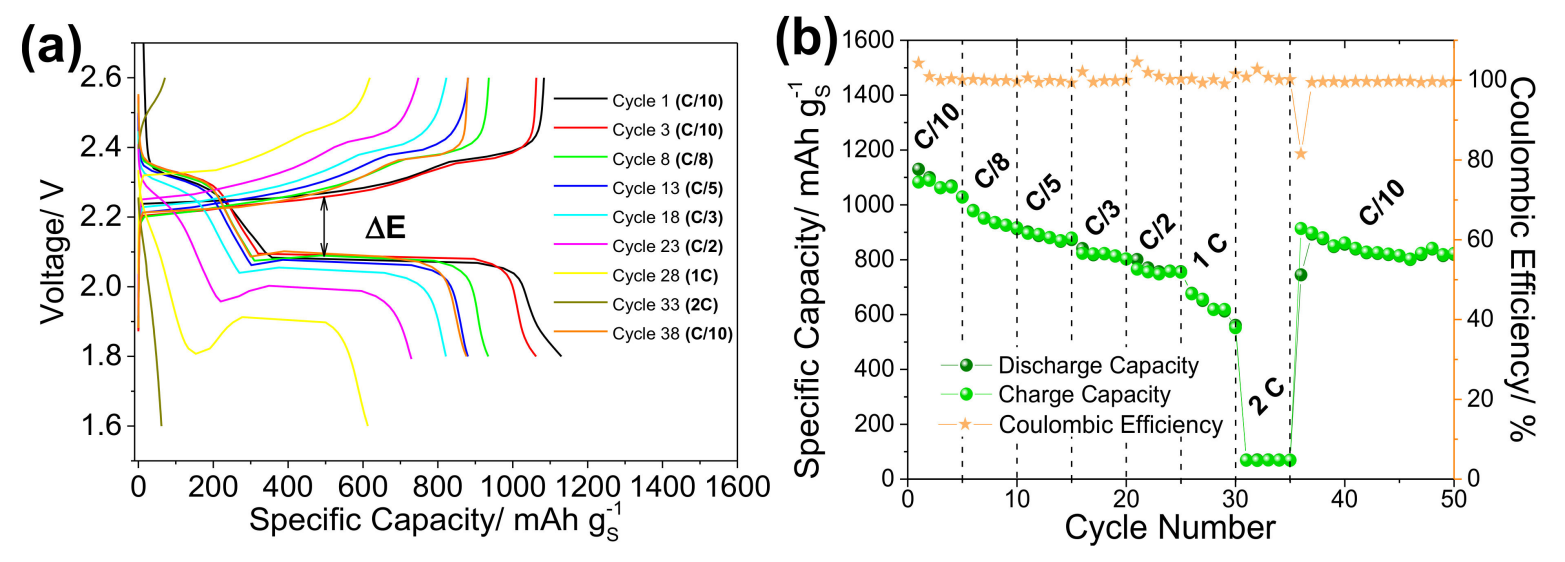
(**a**) Galvanostatic charge/discharge curves at different rates. (**b**) Rate performance.

The study of the performance of the electrode was complemented with measures of rate capability between *C*/10 and 2*C*. A discharge/charge curve at each rate selected as an example is shown in Figure 4a. As expected, a gradual decrease in the delivered capacity was observed with increasing rate (average capacity of 1063, 935, 888, 817, 753, and 646 mAh·g^−1^ at *C*/10, *C*/8, *C*/5, *C*/3, *C*/2, and 1*C*, respectively). In contrast, at 2*C*, the drop was abrupt, and the cell barely delivered 70 mAh·g^−1^ (Figure 4b). The decrease is a consequence of the increased voltage gap (*∆E*) between the charge and discharge processes, parallel to the increase in the rate caused by the sluggishness of the electrochemical process, especially at high rates. *∆E* varied from 0.17 to 0.50 V when changing from *C*/10 to 1*C*. For 2*C*, the value was 0.7 V, which is very close to the voltage window used. Despite the poor performance of the electrode at 2*C*, when the current was lowered back to the pristine value of C/10 during the rate capability test, the electrode maintained a high average capacity of ca. 808 mAh·g^–1^. 

Throughout this section, we have made several comparisons between the behavior of our activated carbon and that recently published by Chen et al. [49]. We have extended this comparison to the electrochemical data collected in Table 1. Apart from the differences in the preparation method, which leads to different textural properties, two differences directly related to the electrochemical response of the electrode must be highlighted: S loading and the voltage window. The S loading of our electrode was almost three times higher. In contrast, the voltage window was almost half less. A higher S content of the electrode would decrease the specific capacity due to the insulating nature of the element, among other reasons. However, the objective of these investigations was to increase the S content so that the delivered energy by the cell would increase. Expanding the voltage window would increase the specific capacity, but in this option, we must be very cautious due to the presence of side reactions, most likely with electrolyte decomposition. In fact, after finishing the low-voltage plateau, the discharge curves included in the reference [49] fell less steeply than that observed in Figure 3a. From 1.8 to 1.5 V, the calculated discharge overcapacity was ca. 200 mAh·g^−1^. Something similar occurred in the charge curve between 2.6 and 3.0 V, detecting an overcharge capacity somewhat lower (70 mAh·g^−1^). Consequently, by making this correction, the capacity values would be lower. Even without this correction, the performance of the electrode made with our activated carbon was better than that obtained by Chen et al. [49], as confirmed by the higher capacity values obtained, not only in the prolonged cycling test (although recorded at different rates) but also in the results of rate capability, which were, in this case, measured at the same current rates. We believe that the cause may lie not only in the different method of preparing the carbon, but also in a higher incidence of other factors related to the electrochemical measurements. It would be very beneficial for the scientific community if we standardized the conditions of the electrochemical measurements, such as the electrode composition, voltage windows, electrolyte concentration, and current collector, to avoid divergent measurements for materials of similar textural and structural characteristics, such as the carbon studied here.

**Figure 5:**
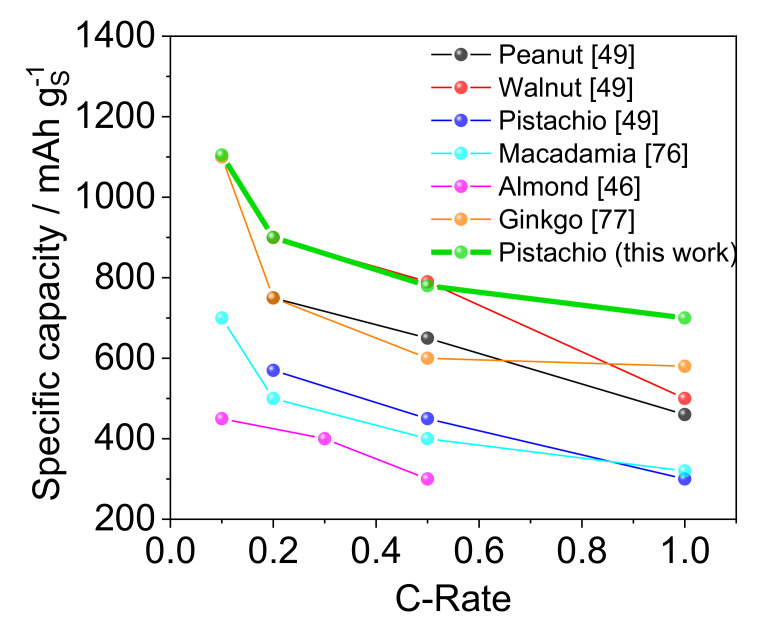
Variation in the specific discharge capacity reported in the literature as a function of the rate.

Finally, capacity values derived from rate capability measurements of carbons obtained from different dried fruit shells, specifically nuts, are collected in Figure 5 [46,49,76,77]. The capacity released by our carbon is among the highest, for both low and high current rates. These results show that carbon from pistachio shells combined with the use of H_3_PO_4_ as an activating agent can be a promising material for future scaled-up Li–S batteries, with an important contribution from circular economy and sustainability models.

## 4. Conclusions

In summary, highly porous carbon as an efficient host material for a sulfur cathode was successfully prepared through H_3_PO_4_-based activation and simple carbonization of pistachio shells. The hierarchical and bimodal micro-mesoporous structure with high specific surface area and large pore volume, as well as residuary functional groups, can significantly limit the shuttle effect of polysulfide through a combination of physical and chemical adsorptions, which enhances sulfur utilization, promotes the reaction kinetics, and allows for remarkable rate capability and long-cycling stability for the assembled Li–S batteries. This work provides a low-cost, eco-friendly, and simple strategy for the preparation of sustainable host materials for high-performance Li–S batteries using natural biomass as a precursor. 

## Figures and Tables

**Table 1:** Selected properties of lithium–sulfur (Li–S) cells made from activated carbons derived from pistachio shells (reported in the literature). The properties of our carbon are also included for comparison.

Preparation Method	*S_BET_* m^2^·g^−1^	S Loading mg·cm^−2^	Long Term Cycling				Rate Capability			Reference
			Voltage Window V	*C_i_*	*C_f_*	Cycle	Rate	Rate	*C_average_* mAh·g^−1^	
				mAh·g^−1^						
Activation with ZnCl_2_, 550 °C S impregnation by melt diffusion (70:20:10) ^1^	1149	0.84	3.0–1.5 ^2^	980	380	200	*C*/5	*C*/5 *C*/2 1*C*	580 450 320	[24]
Activation with H_3_PO_4_, 800 °C S impregnation by wet milling(80:10:10) ^1^	1345	2.5	2.6–1.8	1193	570	300	*C*/10	*C*/5 *C*/2 1*C*	888 753 646	This work

^1^ Electrode composition (composite:carbon black:PVDF ratio). ^2^ Voltage window drawn in the figure. In the text, the indicated voltage window is 1.0–3.0 V.
